# Intact intracranial breast prosthesis: a 28-year CT follow-up after treatment of late hemispherectomy complications

**DOI:** 10.1007/s00381-014-2602-5

**Published:** 2014-12-07

**Authors:** V. Sorano, M. Telesca, F. Pediconi, D. Bova, F. Guidetti

**Affiliations:** 1 Department of Radiological, Oncological and Pathological Sciences, University of Rome “Sapienza”, Viale Regina Elena 324, 00161 Rome, Italy; 2 Department of Radiology, Loyola University Medical Center, Maywood, IL 60153 USA; 3 Ostia Radiologica, C.so Duca di Genova, 26, Ostia Lido, 00121 Rome, Italy

**Keywords:** Silastic, Silicone breast prosthesis rupture, Cerebral hemispherectomy complications, Filling-reduction cranioplasty, Head computed tomography

## Abstract

Anatomical hemispherectomy has had excellent results in treating drug-resistant seizures of infantile hemiplegia. This technique of hemispherectomy consists in the removal of a whole hemisphere, with or without the basal ganglia, the end result being a large cavity left at the end of the operation. The technique, however, is considered to be weighted by important complications, in particular intracranial hemorrhages due to vessels tearing secondary to dislodgement of the remaining hemisphere. Several techniques have been consequently proposed to reduce the volume of the residual hemicranial cavity. An alternative measure is the filling of the cavity itself. We have demonstrated that this type of procedure can be carried out using a silicone breast prosthesis. In this report, we demonstrate also that such an implant can have a surprisingly long duration in its unusual location.

## Introduction

Anatomical hemispherectomy is associated with the best results in controlling medically refractory seizure disorders due to diffuse unilateral epileptogenic lesions. This type of operation, however, has been regarded to be weighted by an excessively high incidence of late complications, such as bleeding in the cavity, hydrocephalus, and superficial hemosiderosis [1]. The last two complications were considered to be the result of chronic intracranial hemorrhages secondary to vascular tearing and lacerations induced by the mechanical dislodgement of the remaining healthy cerebral hemisphere towards the contralateral cavity created by the excision of the pathological hemisphere. The role and even the existence of superficial cerebral hemosiderosis have been challenged by authors who considered the late clinical neurological deterioration, anecdotally reported in patients who had undergone anatomical hemispherectomy, to be rather the effect of a late hydrocephalus [2]. Hydrocephalus could remain easily undetected prior the introduction of computed tomography (CT) [3, 4]. Indeed, superficial hemosiderosis has never been reported in the last four decades [2]. Its presumed occurrence and presumed cause that is the excessive dislocation of the preserved cerebral hemisphere due to the empty intracranial space created by the surgical operation continue to negatively influence the neurosurgical attitude. This in turn prevents patients to benefit of an anatomical hemispherectomy.

The patient of whom we are discussing in the present article successfully underwent an anatomical hemispherectomy in 1969, at the age of five. In 1985, 16 years later, the patient was successfully treated for late complications [5]. Among the other procedures this patient received a Silastic breast implant within the empty hemicranium to limit the mechanical dislocation of the healthy cerebral hemisphere.

After 28 years since implantation, the results of the CT, showing the integrity of the prosthesis, prompted us to this report.

## Case report

**Fig. 1:**
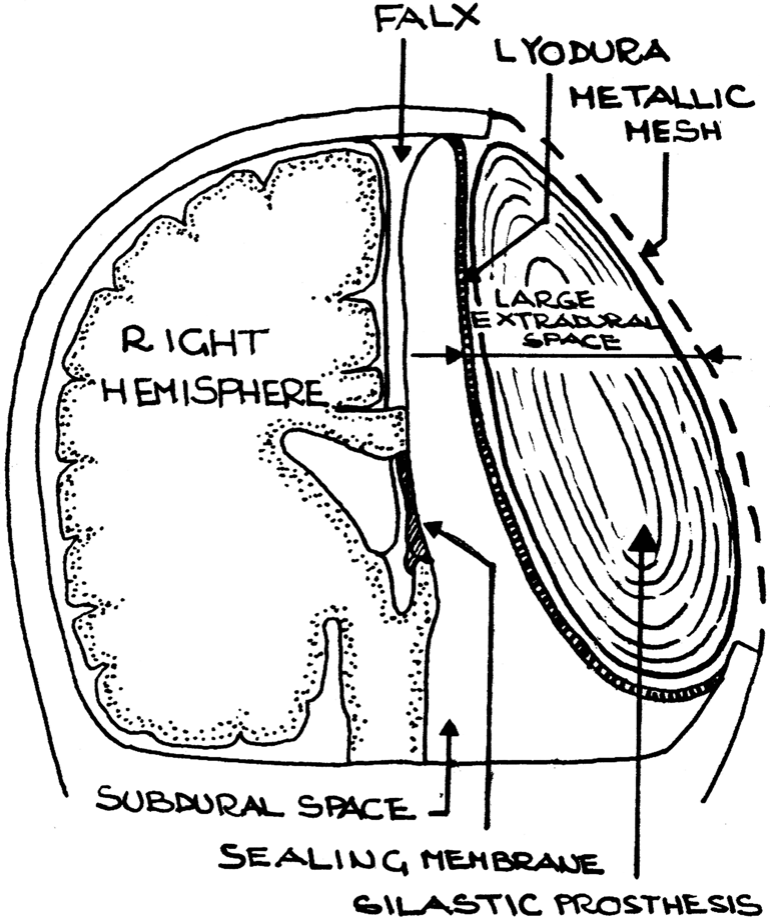
Filling-reduction cranioplasty. The subdural space is reduced with Lyodura, the extradural space with a flattened curvature metallic mesh. The Silastic prostesis fills the remaining extradural space

This 50-year-old man underwent left anatomical hemispherectomy at the age of 5 years because of infantile hemiplegia and intractable seizure disorders. Three months after the operation, the bone flap was removed because of osteitis and substituted by an acrylic cranioplasty in 1971 that is 2 years after the primary operation. The seizures were controlled and the child showed a satisfactory motor and cognitive development, with improvement of the right hemiparesis and good academic results. In 1984, the patient fell off a bicycle with severe head trauma. He developed an epidural hematoma on the right side, bleeding into the left hemispherectomy cavity, and decompensation of a previously documented arrested hydrocephalus [4]. The patient underwent an emergency right craniotomy to remove the epidural hematoma at the Ancona University Hospital and remained for 3 months in the intensive care in a comatous state, with a new left-sided hemiparesis and reoccurrence of seizure disorders. The patient was then referred to San Camillo Hospital in Rome where he underwent further surgical procedures by one of the authors (SV). A temporary external shunt of the right ventricle was applied to wash and clean the hemorrhagic CSF. A fractured acrylic resin cranioplasty was removed. A naturally “reparative” process closing the left foramen of Monroe was respected. A gel-like chronic subdural hematoma of the cavity was removed. A reduction duraplasty, with a Lyodura patch, was performed to reduce the volume of the subdural hemispherectomy cavity and to substitute a thick hemorrhagic dura. A permanent V-P shunt was applied. Subsequently, an intracranial Silastic breast prosthesis was apposed on the Lyodura patch and maintained in place using an overlying metallic mesh with flattened curvature to further reduce the hemicranium (Fig. 1). The rationale for these procedures was previously described in detail in this journal [5].

The patient’s clinical condition progressively improved with recovery of cognitive ability and almost disappearance of the left-sided post traumatic hemiparesis. Several CT control examinations were performed, the last one in 2013, 28 years after the implant. The last examination showed the integrity and the satisfactory survival of the breast silicone implant covered by the metallic mesh with a focal calcification on the anterior aspect of the shell (Fig. 3).

## Imaging

The patient underwent several clinical and CT follow up as MRI was contraindicated due to the presence of old metallic surgical clips. The last CT was performed using standard parameters on a multidetector CT scanner without contrast administration. Multiplanar reconstruction (MPR) and 3D volume rendering (VR) were then obtained from the axial plane acquisitions. The study was compared to several previous CT of the patient.

**Fig. 2:**
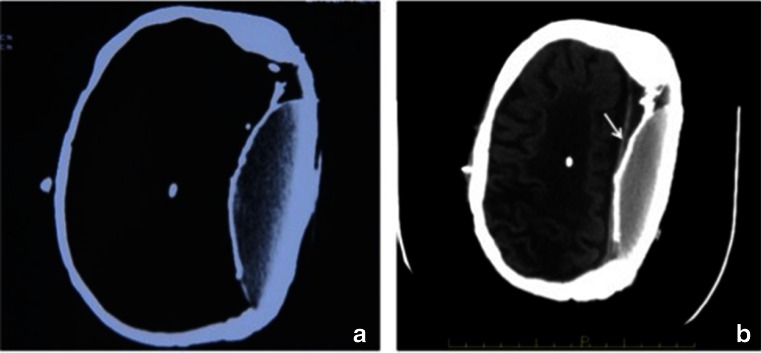
Axial CT from 1996 (**a**) and 2013 (**b**). Both CT show left hemispherectomy and filling-reduction cranioplasty. The left cavity is filled by a breast silicone implant covered by a metallic mesh. Medially to the implant, there is a calcified lyodura. Right V-P shunt is seen. The appearance on the 2013 CT (**b**) is unchanged comparing from the 1996 (**a**)

The CT shows left hemispherectomy and a filling-reduction cranioplasty. The left hemicranium is filled by a breast silicone implant covered by a metallic mesh (Fig. 2).

**Fig. 3:**
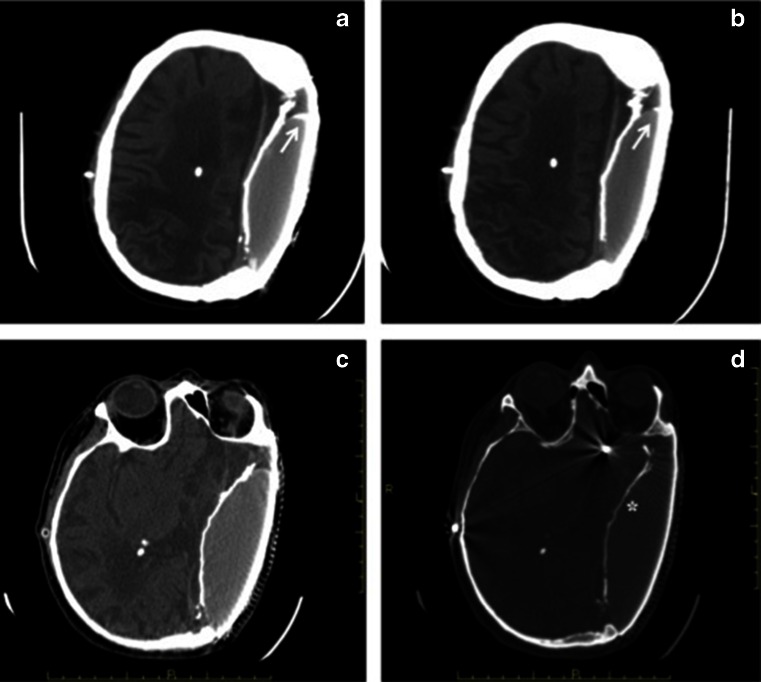
Axial CT with different window levels: soft tissue (**a**–**c**) and bone window (**d**). CT scan shows a white, high attenuation ring, indicating that implant shell is intact. The silicone density is homogeneous and there are no CT signs of rupture, neither wrinkling nor infolding of the implant shell (**a**–**c**). There is also a focal calcification of the capsule on the anterior aspect of the shell (*white arrow*). Both the anterior and posterior margins are smooth with rounded shaped angles with the metallic mesh (**c**). Medially to the implant there is a calcified Lyodura (*asterisk*). Right V-P shunt is seen. Enlargement of the supratentorial ventricular system and prominent subarachnoid space surrounding the right cerebral hemisphere

**Fig. 4:**
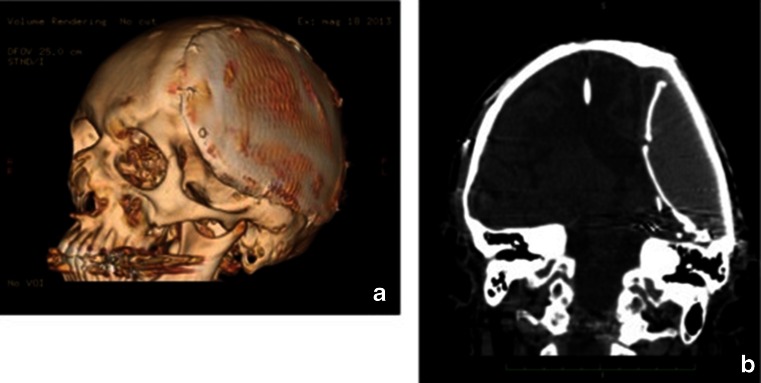
3D VR (**a**) and coronal CT reformat (**b**). Both views demonstrate nearly seamless contour between the tantalium plaque and the native cranial bone with the intended “flattened” cranial contour. The calcified Lyodura is well seen on the coronal view (**b**) as well as the uniform soft tissue density within the implant

The silicone implant demonstrates uniformly greater than soft tissue density, without internal filling defects and no deformity or infolding of the implant shell suggesting rupture of the capsule. The sharply demarcated contours of the Lyodura and of the implant capsule are easily appreciated: the latter is smooth and intact, with a focal calcification of the capsule on the anterior aspect of the shell. Both the anterior and posterior margins are smooth with rounded convex borders in relation to the metallic mesh. The thick calcified rim is composed of closely justaxposed Lyodura and implant shell. Bone windows corroborate the integrity of the implant capsule/Lyodura complex along its calcified portions (Fig. 3d). There is a markedly and diffusely decreased attenuation of the white matter of the fronto-parietal region along with increased sulcal prominence and thinned grey matter in the parietal region, as hallmarks of chronic atrophy. No signs of hydrocephalus or other suggestion of obstruction to CSF flow were noted in the remainder of the study (Fig. 3). The 3D VR images and the coronal view demonstrate nearly seamless contour between the metallic mesh and the native cranial bone, with the intended “flattened” cranial contour (Fig. 4). When compared to a prior CT obtained in 1996, the study doesn’t show any significant difference (Fig. 2).

The most frequent and relevant diagnostic query for breast silicone implants is the evaluation of the integrity. The median life expectancy of normal quality silicone implants is around 10–16 years and is related to the thickness and quality of the elastomeric shell. The most frequent cause of breast implant rupture is the mechanical stress. In our patient, the particular location of the implant, protected by a rigid shell of bone and metallic mesh, markedly decreases the possibility of direct trauma to the implant and protects from any repeated mechanical stress. This most likely represents the reason of such longevity of the implant. At CT, an intact silicone implant is characterized by an oval shape and homogeneous grey density surrounded by a uniformly thin high-density rim. The typical finding of an intracapsular leaking is the “linguine sign,” and some authors demonstrated that CT allows a good evaluation of this sign [5, 6, 7]. In our case, CT examination offered an optimal evaluation of the implant due to the localization in the head. The absence of surrounding soft tissues and the presence of partially calcified implant shell and calcified Lyodura allow an accurate evaluation of the implant integrity. The homogeneous density inside and outside the implant demonstrated the absence of intracapsular silicone leak and peri-implant effusion.

## Discussion and conclusions

Despite the excellent results of anatomical hemispherectomy in the treatment of drug-resistant epilepsy, especially in cases associated with infantile hemiplegia, concern about the vulnerability for the remaining hemisphere was expressed since the beginning; Laine [8] was worried about “l’inquiétant fragilité de nos opérés vis-à-vis de traumatismes crâniens” and remarked on the “rôle of remplissage,” speculating about a stereotactic method of disconnecting the cortex of the diseased hemisphere from the rest of the brain. He also noticed that emptying a hemicranium favors the shifting of the remaining brain, making the most trivial trauma extremely dangerous. Laine also stressed that such accidents involve those patients who, having the best surgical results, enjoy the greatest autonomy. Oppenheimer and Griffith [9] in 1966, describing a syndrome due to hemorrhage in the cavity, suggested that “a possible solution would be to close off the cavity from the ventricles, taking up the space with a biologically inert prosthesis.” Several complications of anatomical hemispherectomy have been reported. Some authors stress the importance of the hydrocephalus as a major factor for the complications [2]. Rasmussen, Adams, and other authors of hemispherectomy [10–19] employed modified techniques. Functional hemispherectomy, hemidecortication, reduction and shunting of the subdural cavity, and even temporary embolization hemispherectomy have been described.

In 1985 Sorano [5], to treat the late complications of the case we are presenting, performed a filling-reduction cranioplasty keeping in mind the Adams technique [10] to reduce the subdural space. A Silastic breast prosthesis was placed over a “non living” Lyodura. A metallic mesh with a flattened curvature was in turn placed above the prosthesis further reducing the operated hemicranium.

The main goal of this technique was avoiding the dislodgement of the cerebral parenchima, by means of the reduction-duraplasty using Lyodura and the epidural placement of the Silastic breast prosthesis. In the same time, this technique recreates an almost normal intracranial mechanical situation taking advantage of the viscoelasticity of the breast implant as well as assuring an external protection using the metallic mesh. The successful long-term outcome of our patient supports the effectiveness of the Sorano’s technique.

A second point of interest is the long duration of the Silastic breast implant. Although the duration of this type of implant is mainly depending on the quality and thickness of the silicone shell, the exceptionally long duration of this breast prosthesis in our patient could have been due also to its specific location that is the limited exposition to mechanical stress compared to the usual thoracic placement and the possible different immunological response.

Dealing with the hemispherectomy patients is always a revealing challenge in many fields, from neuroplasticity to physical aspects. Exceptional patients sometimes suggest and require exceptional approaches. New techniques when validated through accurate long-term follow-up could be applied to other comparable clinical situations.
